# Factors associated with return to meaningful activities following physiotherapy for pelvic girdle pain during pregnancy: 3-year follow-up of a randomised controlled trial

**DOI:** 10.1136/bmjopen-2025-113480

**Published:** 2026-03-18

**Authors:** Annika Svahn Ekdahl, Monika Fagevik Olsén, Annelie Gutke

**Affiliations:** 1 Department of Health and Rehabilitation, University of Gothenburg Sahlgrenska Academy, Gothenburg, Sweden; 2 Department of Physiotherapy, Sahlgrenska University Hospital, Goteborg, Sweden

**Keywords:** Pregnancy, Acupuncture, Physical Therapy Modalities, Postpartum Period, PAIN MANAGEMENT

## Abstract

**Objectives:**

To investigate whether women who experienced pelvic girdle pain (PGP) during pregnancy were able to engage in meaningful activities at 4 months and 3 years post partum, and to identify factors associated with long-term functional outcomes.

**Design:**

Long-term follow-up of a randomised controlled trial comparing acupuncture and transcutaneous electrical nerve stimulation for pregnancy-related PGP.

**Setting:**

Physiotherapy outpatient clinics in Sweden.

**Participants:**

In total, 113 pregnant women with clinically verified PGP were randomised in the original trial; 86 participated in the initial study phase, 77 attended the 4-month follow-up and 57 completed the 3-year follow-up questionnaire.

**Primary and secondary outcome measures:**

The primary outcome was functioning, assessed using the Patient-Specific Functional Scale (PSFS). Secondary outcomes included self-reported PGP, overall functioning and the prognostic value of pelvic pain provocation tests at 4 months post partum for persistent PGP at 3 years.

**Results:**

3 years post partum, the mean PSFS score was 8.64, and 45.6% of the participants scored 10, indicating full return to baseline activities. In repeated linear regression analyses, estimated PSFS scores were approximately 3 points lower at baseline and post-treatment compared with the 3-year follow-up (both p<0.001), indicating improved functioning over time, with no differences between treatment groups. Higher pain-related concern and higher Pelvic Girdle Questionnaire scores were associated with greater activity limitations (estimate −0.21, p=0.019 and −0.06, p<0.001, respectively). Logistic regression showed that the number of positive pelvic provocation tests at 4 months post partum significantly predicted self-reported PGP at 3 years (OR 2.98, 95% CI 1.26 to 7.05, p=0.013).

**Conclusions:**

Most women with pregnancy-related PGP regained functioning by 4 months post partum, and this recovery was sustained at 3 years. The number of positive pelvic provocation tests at 4 months post partum predicted persistent pain at 3 years, suggesting potential prognostic value for identifying women at risk of long-term PGP and informing postpartum follow-up strategies.

**Trial registration number:**

In ‘FoU i Sverige’ (R&D in Sweden) No. 12726. https://www.researchweb.org/is/sverige/project/127261.

**STRENGTHS AND LIMITATIONS OF THIS STUDY**

- The study’s long-term follow-up provides valuable information on sustained effects, which is uncommon in pelvic girdle pain (PGP) intervention research.
- Statistical power at the 3-year follow-up was reduced due to higher attrition in the transcutaneous electrical nerve stimulation group.
- Power calculations were based on the Oswestry Disability Index, a measure not specific to PGP, which may have limited sensitivity to PGP-related changes.
- The predominance of participants with higher education limits the generalisability of the findings to more diverse populations.
- Discrepancies between self-reported and clinically verified PGP highlight potential limitations in symptom assessment.

## Introduction

 Pelvic girdle pain (PGP) is common during pregnancy^1^ and may significantly impact women’s functioning, work capacity and participation in social and leisure activities.^2^ The exact cause of PGP in pregnancy is not fully understood; various theories suggest hormonal changes affecting sacroiliac joint biomechanics and muscular function, and also inflammatory components as plausible causes.^3^

While PGP typically resolves within 6 months post partum for most women,^4^ some continue to experience it for many years after delivery.^5^ However, the reasons behind longstanding PGP remain unclear.^3^ Identified risk factors for postpartum pain include prepregnancy factors, such as a body mass index >25 and a history of lumbopelvic pain, and pregnancy-related factors, including PGP in a previous pregnancy, depressive symptoms and a heavy workload during pregnancy.^6^ Treatment options for PGP include exercises for improving muscle function in the pelvis, trunk and hips, alongside the use of pelvic belts and acupuncture^7^,^9^ or transcutaneous electrical nerve stimulation (TENS).^10^ Despite the absence of an established strategy for preventing PGP, research has suggested potential benefits regarding exercise to prevent lumbar and PGP during pregnancy.^11^ Encouraging physical activity during pregnancy is crucial, offering general health benefits, preserving muscular function^12^ and possibly reducing the risk of pregnancy-related complications such as diabetes, hypertension and pre-eclampsia.^13^

In a previous RCT, our research group investigated the effectiveness of acupuncture and TENS for managing PGP during pregnancy.^10^ After 5 weeks of treatment, no significant differences were found between groups in terms of functioning or other variables. However, both intervention groups maintained physical activity levels and experienced significant reductions in pain intensity and pain-related concerns. It is noted that physical activity levels tend to decrease during pregnancy,^14^ with about 70% of Swedish pregnant women failing to meet recommended activity levels,^15^ a trend that continues post partum.^16^ If the pregnant woman maintains an active lifestyle, it may reduce the severity of PGP and/or lumbar pain both during and after pregnancy.^17^ Nonetheless, PGP often poses a significant barrier to daily activity,^18^ which in turn may have socioeconomic consequences as women with PGP have an increased risk of requiring sick leave compared with healthy pregnant women.^19^ Additionally, they are more likely to experience depressive symptoms, which not only affect their immediate health but also have long-term implications, including lower recovery rates from PGP and a contributing factor to its persistence.^20^

Currently, knowledge is sparse regarding whether treatments for PGP during pregnancy have a lasting impact on women’s long-term functioning, a concept that includes all bodily functions, activities and participation across various contexts and integrates both physical and psychological dimensions.^21^ Thus, functioning includes everyday activities, exercise, work-related activities and social interactions. To the best of our knowledge, no previous study has assessed functioning in postpartum women using measurements derived from activities individually selected by the participants, as the Patient-Specific Functional Scale (PSFS).^22^ Patient-Reported Outcome Measures such as the Pelvic Girdle Questionnaire (PGQ)^23^ often reflect a selection of standardised activities as representative samples rather than capturing the full range of personally meaningful functional limitations and thus, not fully capture the personal impact of PGP. In contrast, PSFS allows individuals to identify and rate activity limitations that are specifically relevant to their daily lives and personal goals. For example, while walking on flat ground may be manageable, walking a dog that pulls on the leash in uneven forest terrain may be difficult—an activity unlikely to be captured by standard measurements. Our aim was to follow whether women who experienced PGP in pregnancy were able to engage in the activities they needed and wanted to perform 3 years post partum, thus necessitating a more person-centred approach to measurement. Therefore, this study aims to, in a cohort of women who participated in an RCT comparing acupuncture and TENS for PGP in pregnancy, investigate functioning, physical activity level and possible remaining PGP at 4 months and 3 years post partum, respectively, investigate relationships between factors that could impact functioning 3 years post partum, resulting from remaining PGP symptoms.

## Methods

### Design

This study is a follow-up conducted at 4 months and 3 years post partum, building on a previous randomised controlled trial (RCT).^10^

### Settings and participants

Women with self-reported PGP in a single fetus pregnancy in gestational week 12–28 recruited through maternity healthcare centres in two Swedish cities and randomised (1:1) into two groups. Women with a history of PGP in a previous pregnancy were not excluded; participants were recruited from the routine clinical flow of the clinic and included both first-time and recurrent cases. All participants (n=113) had PGP verified through a clinical assessment. Exclusion criteria were any previous orthopaedic, systemic or malignant condition affecting the lumbopelvic region, or any obstetric complication that contraindicated physiotherapy. At inclusion, the mean age of the women was 30.8 years, in mean gestational weeks 20.8, and 31% were pregnant for the first time. Details of recruitment and descriptive data are published elsewhere.^10^

### Interventions

A treatment period of 5 weeks with either acupuncture (10 sessions) provided by a physiotherapist or daily home-based TENS. In addition to the assigned intervention, all participants received general written advice about how to manage PGP. The interventions are described in detail in our previous study.^10^

Women who completed the intervention (n=86) were invited to a follow-up visit 4 months post partum and those who attended that visit (n=77) received a follow-up questionnaire by mail 3 years post partum.

### Outcomes

**Figure 1:**
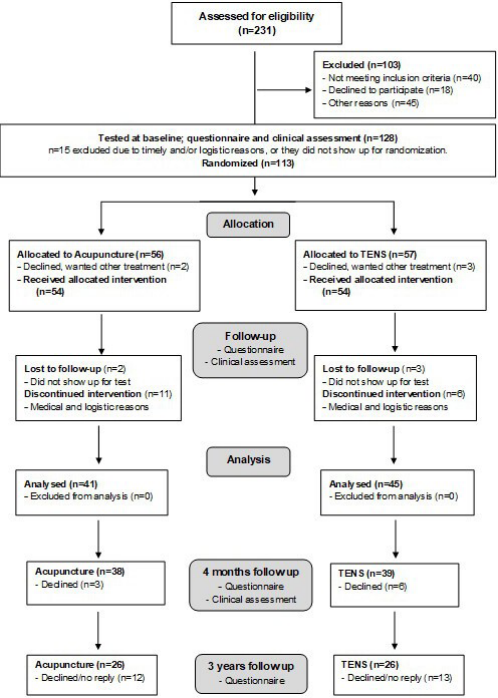
Flow chart of the study from inclusion to 3-year follow-up. TENS, transcutaneous electrical nerve stimulation.

The measurements in the RCT and the 4 months and 3 years follow-ups, respectively, included both questionnaires and clinical assessments at different timepoints, displayed in figure 1.

The clinical assessments were performed to confirm PGP and to exclude the hip and/or lower back with neurologic symptoms as origins of pain,^24^ and the assessment was repeated post treatment and at the 4 months follow-up. The procedure included a neurological examination, test of hip range of motion, pelvic pain provocation tests (MAT-test, P4-test, sacroiliac compression- and distraction test, sacral thrust), a brief mechanical assessment of the lumbar spine, the active straight leg raise test and a unipedal stance test.^10 24 25^ To be classified as PGP, pain should be experienced distally or laterally to the L5/S1 region, over the buttocks, and/or the pubic bone and confirmed by at least two positive pain provocation tests or a positive ASLR test. Additionally, at inclusion, indicated by a score of ≥20% on the Oswestry Disability Index (ODI) and/or a score ≤6 in one self-chosen activity on the PSFS.^10^

The questionnaires contained a pain drawing where participants marked pain location on a female body chart, PROMs for functioning, pain intensity, pain-related concern, physical activity level, work ability, depressive symptoms/anxiety and general health.^10^ In addition, questions were asked about height/weight, parity, educational level, if the woman managed financially, sick leave, use of analgesics, delivery mode and eventual complications. In the follow-up questionnaires, questions about treatment satisfaction and possible side effects were included.

The main outcome of the current study was the PSFS,^22^ an instrument where the individual woman, at inclusion, chose two activities that were troublesome for her because of PGP. The activities were graded on an NRS where 0 corresponds to inability to perform the activity and 10=she could perform the activity unrestrictedly or as before the onset of PGP. The two activities chosen by the individual women at inclusion were the same ones used throughout the study. The PSFS is reliable and easy to use for a diversity of musculoskeletal conditions.^22^

**Figure 2:**
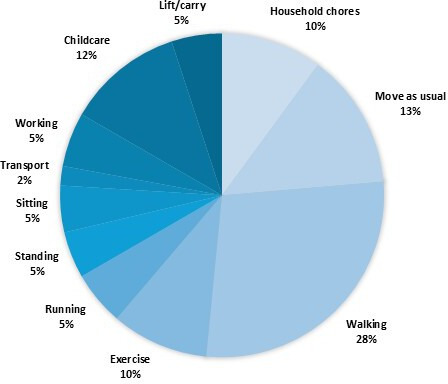
Distribution of participants’ self-chosen PSFS activities. PSFS, Patient-Specific Functional Scale.

To provide an overview of the different PSFS activities the participants chose and graded as troublesome, they were grouped into categories (figure 2). Most of these activities ranged from light to moderate intensity. However, the intensity of a given activity could vary depending on how it was performed. For example, walking may be classified as light, moderate or even vigorous, depending on the pace and environment. Similarly, transportation-related activities could span from sedentary (eg, riding a bus or train), to light (eg, driving a car), to moderate or vigorous (eg, cycling to work). Household chores and childcare tasks also exhibit considerable variability in intensity.^26 27^ Given the wide variation in nature and intensity of the chosen activities, the categories were not included as variables in the regression analyses.

### Statistical analysis

Data were analysed using SPSS V.28 (IBM). For descriptive statistics, mean (SD) and frequency (percentage) were used. Independent t-test and χ²-square test were used to compare data between the two intervention groups.

A power analysis was conducted prior to the initiation of the RCT to determine the appropriate sample size. The calculation was based on the expected effect size and variability of the primary outcome, functioning/disability, as measured by the ODI, from baseline to the post-treatment period (6 weeks after inclusion).^10^ According to this calculation, a sample size of 30 women/group was required for the 3 years follow-up. The same outcome measures for functioning (PSFS and PGQ-Total Score, PGQ-T) were used at all time points (baseline, 4 months and 3 years). Although the initial power analysis did not account for potential differences in effect sizes at these follow-up points, we addressed this in our statistical model.

It was hypothesised when the initial RCT was designed that the interventions might have different long-term effects, as acupuncture required clinical visits whereas TENS could be used independently at home; therefore, between-group differences were calculated in the analyses and kept throughout the follow-ups.

We applied a repeated linear regression model with an unstructured covariance structure to analyse all available data for the variables of interest from baseline through to the 3-year follow-up. This approach allowed us to account for correlations between repeated measurements across time points, investigate change over time within and between the intervention groups, maximising the use of available data despite attrition.

**Figure 3:**
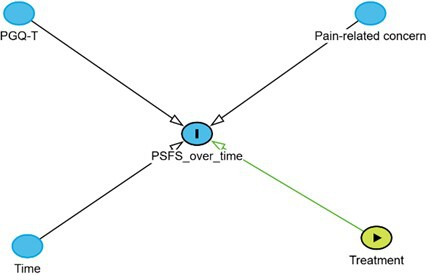
Directed acyclic graph illustrating the assumed causal structure of variables in the repeated linear regression model. PGQ-T, Pelvic Girdle Questionnaire Total Score; PSFS, Patient-Specific Functional Scale.

All available data were used for the participants at each timepoint with PSFS^22^ as the dependent variable. Independent variables were selected a priori, based on clinical reasoning including previously identified factors, measured in pregnancy, that may impact the level of functioning due to PGP. The result of the initial RCT showed that pain-related concern was reduced at follow-up,^10^ and emotional distress is a factor that may contribute to PGP in pregnancy and post partum.^6^ PGQ-T was used to measure limitations in functioning and symptom severity including pain intensity, and the instrument is a valid and reliable instrument for PGP.^28^ The final model included treatment group, time point (with the 3-year follow-up specified as the reference category), pain-related concern and PGQ-T as fixed effects, as well as interaction terms between treatment group and time point and between pain-related concern and PGQ-T. The assumed causal relationships were specified a priori and are illustrated in a directed acyclic graph (figure 3). Pain-related concern and PGQ-T were treated as prognostic factors influencing functional outcome (PSFS). These variables were measured prior to the intervention and during follow-ups. No post-randomisation mediators were adjusted for.

No multicollinearity was detected between the chosen variables. The variables and residuals were checked for linearity, homoscedasticity and normal distribution of residuals without any concerns, and thus the conditions for the model were met.

A simple logistic regression was used to assess whether the number of positive pelvic provocation tests (summarised for each participant; 0–6) at 4 months post partum might predict remaining PGP (self-reported yes/no) at 3 years. Logistic regression assumptions were checked and met; the dependent variable was binary, and the predictor was continuous with acceptable distribution. Multicollinearity was not a concern.

### Patient and public involvement

No patients or members of the public were involved in design, conduction or interpretation of the study.

## Results

No statistically significant differences between treatment groups were observed for any of the variables at the 4-month or 3-year follow-up. At the 4-month follow-up, 68.8% of participants reported pain in the pelvic region, while 52.1% met the clinical criteria for PGP. At 3 years post partum, 61% of women reported similar pain (table 1).

**Table 1:** Descriptive data from inclusion to 3-year postpartum follow-up, including clinical tests to verify PGP

Variable	Baseline (n=113)	6 weeks follow-up (n=86)	4 months post partum (n=77)	3 years post partum (n=57)
Self-reported PGP; yes, n (%)	113 (100)	84 (98.8) n=85	53 (68.8)	35 (61.0)
Clinically verified PGP, n (%)	113 (100)	78 (91.8) n=85	38 (52.1) n=73	n.a.
BMI, mean (SD)	26.03 (4.22)	27.57 (4.37)	25.20 (4.03) n=*75*	24.96 (4.70) n=*54*
Parity, n (%)		n.a.	n.a.	
0	43 (38.1)			0
1	58 (51.3)			12 (21.1)
2	11 (9.7)			35 (61.4)
3	1 (0.9)			10 (17.5)
Work ability NRS, mean (SD)	6.44 (2.38)	5.51 (2.81)	8.45 (1.91)	9.05 (1.55)
Educational level, n (%)		n.a.	n.a.	n.a.
Elementary/high school	78 (69.0)			
College/university	35 (31.0)			
Manage financially; yes, n (%)	107 (94.7)	79 (91.9)	65 (84.4)	57 (100)
Recommended PA; yes, n (%)	27 (23.9)	17 (19.8)	38 (49.4)	29 (50.9)
Vaginal delivery; yes, n (%)			68 (88.3)	
EPDS score, mean (SD)	7.80 (4.55)	6.21 (4.81)	4.96 (4.38) n=75	n.a.
HADS Depression, mean (SD)	n.a.	n.a.	n.a.	3.65 (2.84)
HADS Anxiety, mean (SD)	n.a.	n.a.	n.a.	2.40 (2.36)
EQ VAS, mean (SD)	62.77 (16.74)	63.49 (17.98)	78.32 (14.49)	77.63 (13.47)
Pain intensity NRS, mean (SD)	6.71 (1.76) n=112	5.45 (2.31)	2.16 (2.62)	2.05 (2.39) n=56
Pain-related concern NRS, mean (SD)	4.42 (2.39)	2.53 (2.16)	1.44 (2.19)	1.25 (1.97) n=56
PSFS NRS, mean (SD)	2.99 (1.51)	3.38 (2.06)	8.19 (2.25)	8.64 (2.05) n=56
PGQ-T, mean (SD)	48.14 (15.45) n=111	47.83 (16.45) n=85	10.15 (12.59)	9.68 (14.26)
Had another baby; yes, n (%)	n.a.	n.a.	n.a.	12 (21.4) n=56
Pregnant at follow-up; yes, n (%)	n.a.	n.a.	n.a.	4 (7.1)
GRC, mean (SD)	n.a.	n.a.	8.62 (1.66)	8.87 (1.21) n=53
Mat test positive; yes, n (%)*	54 (48.2) n=112	50 (58.8)	9 (18.1) n=48	
4P positive; yes, n (%)*				
Unilateral	25 (22.1)	17 (20.0)	11 (27.5)	*n.a*.
Bilateral	80 (70.8)	63 (74.1)	10 (25.0) n=40	
SIJ separation test positive; yes, n (%)*	65 (57.5)	57 (67.1)	4 (10.8) n=37	*n.a*.
SIJ compression test positive; yes, n (%)*	33 (29.2)	17 (20.0)	1 (3.0) n=33	*n.a*.
Sacrum ventral test positive; yes, n (%)*	53 (46.9)	37 (43.5)	4 (12.9) n=31	*n.a*.

All variables are not used at each time point.

* If the woman reported no symptoms, that is, said she was fully recovered at the 4 months postpartum follow-up, no pelvic pain provocation tests were performed. For pain localised over the SIJ, two positive tests (out of five) are required to verify PGP.

† After data collection was completed, we were informed that the Swedish PGQ^41^ differs from the original questionnaire^23^ regarding one item. In the Swedish version, the time frame in item no. 8 is 10 min compared with 60 min in the original version.

ASLR, active straight leg raise; BMI, body mass index; EPDS, Edinburgh Post-Natal Depression Scale; EQ VAS, EuroQoL Visual Analogue Scale; GRC, Global Rating of Change scale; HADS, Hospital Anxiety and Depression Scale; n.a, not applicable; NRS, Numeric Rating Scale; 4P, Posterior Pelvic pain Provocation test; PA, physical activity; PGP, pelvic girdle pain; PGQsve, Pelvic Girdle Questionnaire–Swedish version; PGQ-T, Pelvic Girdle Questionnaire Total Score; PSFS, Patient-Specific Functional Scale; SIJ, Sacroiliac joint.

Approximately 50% of the participants met the WHO guidelines for recommended levels and intensity of physical activity. Self-assessed work ability was high (mean 9.05 of 10) at the 3 years follow-up, 69% of the participants at inclusion had a higher education (college/university), and the majority (>80%) reported managing well financially (table 1). 12 women reported that they had another baby after the completion of the initial RCT and four women were pregnant at the time of the 3 years follow-up. None of the participants presented with depressive symptoms requiring further clinical intervention.

The PGQ-T scores at 4 months and 3 years post partum, respectively, indicated low disability due to PGP, and the PSFS scores showed that the women over time regained ability to perform the activities they chose at baseline. Mean PSFS scores at 4 months post partum were 8.19, increasing slightly at the 3 years follow-up to 8.64 (table 1). At 4 months, 29 women (37.6%), and at 3 years, 26 women (45.6%) scored 10 on PSFS, indicating that they could perform the chosen activities as before debut of PGP. Mean scores on the GRC were 8.62 and 8.87 at these two follow-ups, respectively.

**Figure 4:**
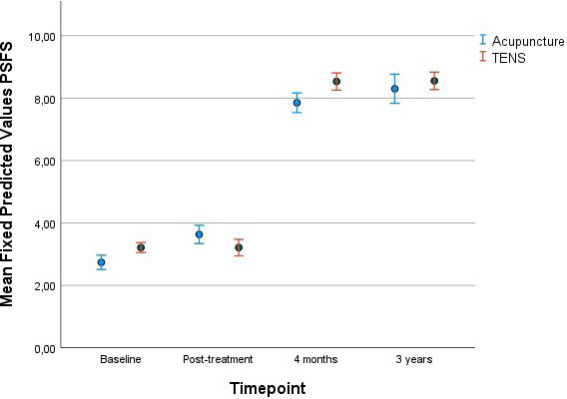
Unadjusted means for the Patient-Specific Functional Scale (PSFS) from the repeated linear regression analysis at the four different time points; baseline, post-treatment, 4 months post partum and 3 years post partum. Error bars: 95% CI. TENS, transcutaneous electric nerve stimulation.

Figure 4 shows the unadjusted mean PSFS scores over the study period, illustrating an overall improvement in functioning from baseline to the 3-year follow-up, with similar trajectories in the acupuncture and TENS groups. No significant differences between treatment groups were observed at any time point.

Results from the repeated linear regression model are presented in table 2. Time was entered as a categorical variable with the 3-year follow-up specified as the reference category. After adjustment for pain-related concern, PGQ-T and their interaction, PSFS scores were significantly lower at baseline and post-treatment compared with the 3-year follow-up (estimate=−2.89 and −2.93, respectively; both p<0.001), indicating lower functioning at these earlier time points. Higher pain-related concern and higher PGQ total scores were both significantly associated with lower PSFS scores (estimate=−0.21, p=0.019 and estimate=−0.06, p<0.001, respectively), indicating greater functional limitations with increasing concern and disability. A small but statistically significant interaction between pain-related concern and PGQ scores was observed (estimate=0.004, p=0.034), suggesting that the association between concern and functioning varied depending on the level of perceived disability.

**Table 2:** Repeated linear regression analysis of factors associated with mean PSFS score

Fixed effects	Estimate	P value	95% CI
Intercept	9.16	<0.001	8.45 to 9.87
Treatment group (reference: TENS)			
Acupuncture	0.09	0.854	−0.91 to 1.09
Time (reference: 3-year follow-up)			
Baseline	−2.89	<0.001	−3.92 to −1.86
Post-treatment	−2.93	<0.001	−3.97 to −1.89
4 months post partum	0.05	0.89	−0.69 to 0.79
Pain-related concern	−0.21	0.019	−0.39 to −0.04
PGQ-T total score	−0.06	<0.001	−0.08 to −0.05
Pain-related concern×PGQ-T	0.004	0.034	0.00 to 0.007
Time×treatment group interactions			
Baseline×acupuncture	−0.49	0.437	−1.73 to 0.75
Post-treatment×acupuncture	0.26	0.684	−1.00 to 1.52
4 months post partum×acupuncture	−0.49	0.376	−1.58 to 0.60

Estimates represent adjusted differences in mean PSFS scores relative to the reference categories (TENS group and 3-year follow-up).

Dependent variable: mean PSFS score.

PGQ-T, Pelvic Girdle Questionnaire Total Score; PSFS, Patient-Specific Functional Scale; TENS, transcutaneous electric nerve stimulation.

Logistic regression was used to assess whether The number of positive pelvic pain provocation tests at 4 months post partum predicted self-reported PGP (yes/no) at 3 years. The model was statistically significant (χ² (1)=8.89, p=0.003), explained 20.2% of the variance (Nagelkerke R²), and correctly classified 67.3% of cases. The number of positive tests was significantly associated with the odds of reporting pain at 3 years (B=1.092, SE=0.440, Wald=6.159, p=0.013. Each additional positive test 4 months post partum increased the odds of reporting pain at 3 years nearly threefold (OR=2.98, 95% CI 1.26 to 7.05, p=0.013).

## Discussion

This long-term follow-up of an RCT showed that participants regained functioning over time, which is consistent with previous research indicating that PGP usually resolves within 3–6 months post partum.^4^ At the 4 months follow-up, 37.6% of the participants reported ability to return to their self-reported meaningful activities (PSFS score of 10), compared with 45.6% at 3 years post partum. This finding may reflect not only residual limitations but also broader changes in lifestyle following childbirth. It is possible that the women were satisfied without returning to their exact prepregnancy activities, or that they adapted by modifying the intensity of activities, substituting activities or redefining what was meaningful in the context of parenting young children. Although not all women reported a complete return to their self-chosen activities, GRC scores at both follow-ups may indicate substantial recovery compared with when they received physiotherapy for PGP. As the PSFS is based on self-selected activities, relevance of the originally chosen activities may also have diminished over time, which could influence reported scores. The observation that levels of functioning were similar at both 4 months and 3 years post partum raises the question of whether PSFS scores at 4 months post partum could be useful in predicting longer-term functional outcomes.

Another important finding was that a higher number of positive pelvic provocation tests at 4 months post partum was associated with an increased likelihood of persistent pain at 3 years, with each additional positive test nearly tripling the odds. This finding supports the potential value of early postpartum assessment for identifying women at risk of long-term pain. Offering a structured physiotherapy follow-up at 4 months post partum may be a feasible strategy to identify women in need of additional support. A standardised cluster of objective clinical tests, which are feasible to perform at a postpartum follow-up, could aid in identifying individuals at increased risk of persistent PGP. Tailored physiotherapy interventions, based on clinical presentation, individual needs and functional limitations, may improve recovery, promote physical activity and support general health.

Mean PSFS scores improved significantly over time and were associated with pain-related concern and PGQ scores, while no differences were found between treatment groups. Although statistically significant, the observed effects were below the minimal important change threshold of 1–3 points for PSFS, suggesting limited clinical relevance.^22^ The women in this study generally reported low levels of pain-related concern at the follow-ups. Nevertheless, pain-related concern remains an important aspect of PGP, as it may contribute to fear-avoidance behaviours that increase the risk of persistent pain.^29^ It is important to distinguish pain-related concerns from clinical conditions like anxiety or depression; worry is a normal cognitive response, not a diagnosis. Women with PGP often report concerns related to symptom progression, physical capacity during childbirth, the ability to care for their child and hesitancy towards future pregnancies due to prior difficulties.^30^ These concerns may stem from limited knowledge about PGP, its expected course and suboptimal pain management strategies.^30^,^32^ Recognising each woman’s unique context, including potentially maladaptive beliefs related to PGP, is essential for delivering individualised and effective interventions.

The significant association between PGQ and PSFS scores may suggest some overlap in the constructs captured by these instruments. However, while the PGQ focuses on commonly affected activities in women with PGP, the PSFS offers a more person-centred assessment by capturing activities that are individually meaningful. This characteristic makes the PSFS particularly valuable for tailoring interventions and for use in clinical practice. As PGP is more commonly associated with pain during movement than at rest,^33^ measures of pain intensity alone may not adequately capture the functional impact of the condition.

Functioning is a multidimensional concept and may be understood differently by different individuals, making it challenging to measure. Standardised questionnaires such as PGQ^23^ may not capture activity limitations that are specific and meaningful to the individual. For this reason, the PSFS was selected as the primary outcome measure. When participants identified problematic activities, these often involved complex everyday tasks such as walking the dog, sitting on the floor to play with a child, or going to the gym. The PSFS is easy to administer in clinical settings, is reliable and could be used in a variety of musculoskeletal disorders.^22^

Physical activity levels in this study were self-reported using two questions addressing the frequency of moderate- and high-intensity activity.^34^ At both follow-ups, approximately 50% of the participants met general physical activity recommendations.^12^ However, physical activity may have been underestimated, as participants may have reported only structured exercise and not everyday activities that contribute to total activity levels. National data from 2021 indicate that approximately 68% of Swedish women aged 30–44 years achieve sufficient levels of physical activity,^35^ suggesting that the women in this study may have been less active than the general population. Identifying pregnant or postpartum women with PGP and insufficient physical activity is important, as sedentary behaviour combined with pain at 4 months post partum has been associated with persistent lumbopelvic pain at 10 months post partum in first-time mothers.^36^ Furthermore, evidence suggests that engaging in at least 80 min of moderate-intensity physical activity per week within 12 weeks post partum may reduce postpartum depressive symptoms.^37^ Although accelerometer data indicate that postpartum women may accumulate sufficient total activity, bouts of moderate-intensity activity are often short and infrequent and may not reach recommended intensity thresholds.^16^

### Strengths and limitations

A major strength of this study is the long-term follow-up, which is uncommon in intervention studies of PGP. However, several limitations should be considered. Statistical power was reduced at the 3-year follow-up due to higher attrition in the TENS group. In addition, the sample size calculation was based on the ODI, as the PGQ was not available at the time of study design.^23^ As ODI was developed for low back pain and may not fully capture PGP-specific limitations,^38^ the use of a different outcome measure could have influenced the power estimation.

Most participants had a high level of education and reported that they managed well financially, which may limit generalisability to other socioeconomic groups. Higher education is associated with higher physical activity levels^39^ and more favourable outcomes in low back pain.^40^

At 4 months post partum, a discrepancy was observed between self-reported symptoms and clinically verified PGP, with not all women reporting pain (68.8%) meeting clinical diagnostic criteria (52.1%). Such discrepancies may reflect mild or nonspecific symptoms captured through self-report measures that may not warrant clinical diagnosis or intervention.^33^ The interpretation of the female body chart used to identify pain location may also have contributed, as the chart includes areas beyond those specific to PGP. Additionally, some women may include pelvic floor-related symptoms when reporting pain in this region.

16 participants had subsequent pregnancies during follow-up; however, recurrence of PGP was not systematically recorded. Therefore, pain reported at 3 years may reflect persistent symptoms from the original pregnancy or new-onset or recurrent PGP related to a later pregnancy.

## Conclusions

This study demonstrates that most women treated with either acupuncture or TENS for PGP in pregnancy had regained functioning by 4 months post partum, and that this recovery was maintained at the 3-year follow-up. However, women with multiple positive pelvic provocation tests at 4 months were more likely to report persistent pain 3 years later. These findings underscore the importance of postpartum follow-up to identify women who have not fully recovered and may benefit from targeted interventions. The use of person-centred outcome measures such as the PSFS may support more individualised management strategies aimed at preventing persistent symptoms and long-term functional limitations.

## Data Availability

Data are available on reasonable request.
