# Supplementary material for: Factors associated with return to meaningful activities following physiotherapy for pelvic girdle pain during pregnancy: 3-year follow-up of a randomised controlled trial
Source: BMJ Open. 2026 Mar 18;16(3):e113480. doi: 10.1136/bmjopen-2025-113480 (PMC13007068; doi:10.1136/bmjopen-2025-113480)
Supplement: Supplementary file 1 [file bmjopen-2025-113480.reviewer_comments.pdf]

PEER REVIEW HISTORY

BMJ Open publishes all reviews undertaken for accepted manuscripts. Reviewers are asked to complete a checklist review form and are provided with free text boxes to elaborate on their assessment. These free text comments are reproduced below.

ARTICLE DETAILS

Title (Provisional)

Factors associated with return to meaningful activities following physiotherapy for pelvic girdle pain during pregnancy: three-year follow-up of a randomised controlled trial.

Authors

Svahn Ekdahl, Annika; Fagevik Olsén, Monika; Gutke, Annelie

VERSION 1 - REVIEW

**Reviewer**	**1**
**Name**	**Magann, Everett**
**Affiliation**	**UAMS Medical Center, Obstetrics & Gynecology**
**Date**	**21-Dec-2025**
**COI**	**None**

In the abstract, Results, I think it would be more meaningful instead of saying the women regained the ability to perform base line activities would give the number, how many out of the 86 regained that ability by 4 months postpartum . Would also give the numbers for higher pain concerns and the higher PGQ scores on questionnaire, What was the number of women who had pelvic provocation tests at 4 months and at 3 years.

Introduction: line 19, in the sentence that say "Risk factors for postpartum pain…, line 21: a history of lumbopelvic pain – is that pain she had during pregnancy or a history of lumbosacral pain outside of pregnancy : then the next risk factor is PGP in pregnancy which makes no since , do you mean in the ongoing pregnancy, are there women who have PGP that starts in the postpartum period,- I am unaware of PGP that starts in the postpartum time frame

In the methods section did you exclude women who had PGP in a prior pregnancy?

In the results, you stated that some of the women because pregnancy again, I am not aware of any studies that have stated the reoccurrence rate of PGP in a subsequent pregnancy. Even though you number are small did you record whether they had a reoccurrence in

subsequent pregnancy? It looks like you could report that for sure on 12 women and maybe as many as 16 using the 3 year follow up.

Results and discussion: in the discussion you make a number of statements about the follow up but your number are not separately given in the text of the result, abstract and are somewhat challenging to extract from your tables

Would like to see in your results or in your discussion How many of the women has a score of 10 at 4 months and at 3 years on the PSFS activities

How many of the women has a positive pelvic provocation at 4 months ?

Page 14 line 45 " at both follow ups 50% of the participants", this is the information that the reader wants and can understand rather than having to search thru tables

---

**Reviewer**	**2**
**Name**	**Kristiansson, Per**
**Affiliation**	**Uppsala Universitet, Public Health and Caring Sciences**
**Date**	**23-Dec-2025**
**COI**	**None**

Review comments to the manuscript "From pregnancy to three years after – maintenance of functioning and physical activity level among women who received physiotherapy for pelvic girdle pain in pregnancy"

An important approach to study the development of pregnancy-related pelvic girdle pain up until three years postpartum. In the present study a cohort of women included in a previous RCT are followed-up. Validated and reliable instruments were used for exposure and outcome measures and adequate statistical analyses were made.

My reflections:

The attrition rate was substantial, about 50%: 113 women were included and 57 were followed-up 3 years postpartum.

My suggestion is to present a flow chart describing the whole passage of time.

Statistical analyses:

Regarding the adjustment for confounders. I wish that could be more clearly presented, since it is not clear to me if the adjustment made also meant an adjustment for mediators. Could a directed acyclic graph be presented?

Results:

Table 1. I find the presentation of differences between treatment groups related to the former RCT not relevant for the present study aims. I would like the Table 1 to be changed to

show the results presented for the whole cohort from study inclusion, post-treatment, 4 months postpartum and 3 years postpartum, regardless of previous treatment group. This will also give the possibility to compare possible differences between the women initially included in the study and the women remaining in the study at the different time points.

Moreover, 60% of the included women had given birth before. To get a wider picture of the cohort I would like to know how many births they had before, not just no/yes.

In paragraph 2 of the Results the different PSFS activities are presented. I believe this section should be moved to the methods section. Instead, the development of PSFS-results across the study period would be presented, similar to the presentation of the PGQ-T scores.

Paragraph 3 of the Results. The differences between the intervention groups I found not relevant for the present study aims. Also, I wonder about the relevance to present incontinence-related symptoms.

The repeated linear regression model:

Why calculated differences between the previous treatment groups? The interesting information is the description of the development of PSFS scores, pain-related concern and PGQ-T scores across the study period. A thought is what does the value -2.89 mean, both the negative value and then also the absolute value? From the methods section I got the information that PSFS is scored between 0 and 10. Also, regarding Table 2, what does the use of square brackets mean?

The logistic regression analysis.

Good that the whole cohort was used in the analysis. To facilitate the interpretation the logistic regression a presentation of the 6 different pain provocation tests and the outcome (no pain/pain) could be presented in a table.

Discussion

My overall point of view is to present the discussion according to the STROBE checklist. I would also that the presentation of results in the discussion is used to a minimum.

VERSION 1 - AUTHOR RESPONSE

Reviewer: 1
Prof. Everett Magann, UAMS Medical Center
Comments to the Author:

In the abstract, Results, I think it would be more meaningful instead of saying the women regained the ability to perform base line activities would give the number, how many out of the 86 regained that ability by 4 months postpartum . Would also give the numbers for higher pain concerns and the higher PGQ scores on questionnaire, What was the number of women who had pelvic provocation tests at 4 months and at 3 years.

Reply: *We have revised the results section of the abstract to make it clearer (p. 2) in line with revisions done in the Results (p. 11) and Discussion section (p. 13).*
Table 1 has been revised and includes the pelvic provocation tests (p. 10).

Introduction: line 19, in the sentence that say "Risk factors for postpartum pain…, line 21: a history of lumbopelvic pain – is that pain she had during pregnancy or a history of lumbosacral pain outside of pregnancy : then the next risk factor is PGP in pregnancy which makes no since , do you mean in the ongoing pregnancy, are there women who have PGP that starts in the postpartum period,- I am unaware of PGP that starts in the postpartum time frame

Reply: *We agree that section of the Introduction was unclear, and it was revised accordingly on p. 4.*

In the methods section did you exclude women who had PGP in a prior pregnancy?

Reply: *No, women with a history of PGP in a previous pregnancy were not excluded. Participants were recruited from the routine clinical flow of the physiotherapy unit, and both women experiencing PGP for the first time and those with a history of PGP in a prior pregnancy were eligible for inclusion. We have clarified this in the Methods section, p. 6.*

In the results, you stated that some of the women because pregnancy again, I am not aware of any studies that have stated the reoccurrence rate of PGP in a subsequent pregnancy. Even though you number are small did you record whether they had a reoccurrence in subsequent pregnancy? It looks like you could report that for sure on 12 women and maybe as many as 16 using the 3 year follow up.

Reply: *In the baseline questionnaire, participants were asked whether they had experienced lower back and/or pelvic pain before the pregnancy during which they were enrolled in the RCT; however, we did not specifically ask about pain during previous pregnancies. At the three-year follow-up, participants were asked whether they had experienced similar pain during the preceding four weeks as at enrolment. Four women were pregnant at follow-up and 12 had given birth again since the RCT. We did not specifically record whether these women experienced recurrent PGP during subsequent pregnancies. Therefore, we cannot determine whether pain reported at three years reflected persistent PGP from the original pregnancy or new-onset or recurrent PGP related to a later pregnancy. As such, the recurrence rate of PGP in subsequent pregnancies cannot be estimated from our data. Information has been added to "Strengths and limitations" at p. 15.*

Results and discussion: in the discussion you make a number of statements about the follow up but your number are not separately given in the text of the result, abstract and are somewhat challenging to extract from your tables

Reply: *We have revised both the Results and the Discussion section and added information for increased readability (p. 9-12, 13-15).*

Would like to see in your results or in your discussion How many of the women has a score of 10 at 4 months and at 3 years on the PSFS activities

Reply: *At 4 months, 29 women scored 10 at PSFS compared to 26 women at 3 years. This information has been added to the results section (p. 11) and discussed on p. 13.*

How many of the women has a positive pelvic provocation at 4 months?

Reply: *We agree that this is valuable information, and it is now added to Table 1 (p. 10).*

Page 14 line 45 "at both follow ups 50% of the participants", this is the information that the reader wants and can understand rather than having to search thru tables

Reply: Thank you for this comment, the discussion has been revised to improve overall clarity p. 13-15.

Reviewer: 2
Dr. Per Kristiansson, Uppsala Universitet
Comments to the Author:

Review comments to the manuscript "From pregnancy to three years after – maintenance of functioning and physical activity level among women who received physiotherapy for pelvic girdle pain in pregnancy"
An important approach to study the development of pregnancy-related pelvic girdle pain up until three years postpartum. In the present study a cohort of women included in a previous RCT are followed-up. Validated and reliable instruments were used for exposure and outcome measures and adequate statistical analyses were made.

The attrition rate was substantial, about 50%: 113 women were included and 57 were followed-up 3 years postpartum.
My suggestion is to present a flow chart describing the whole passage of time.

Reply: *A flow chart of the whole process from RCT to three-year follow up (Figure 1) is added to the manuscript for increased clarity of the process (insert at p. 6).*

Statistical analyses:
Regarding the adjustment for confounders. I wish that could be more clearly presented, since it is not clear to me if the adjustment made also meant an adjustment for mediators. Could a directed acyclic graph be presented?

Reply: *We agree that the distinction between confounders and mediators should be made more explicit.*

The covariates included in the model (pain-related concern and PGQ-T) were selected a priori based on clinical reasoning and previous literature as baseline factors associated with functional limitation in PGP. These variables were measured at inclusion in the RCT and at all follow-ups

The model was not adjusted for post-randomization mediators of the treatment effect.

We acknowledge that pain-related concern may also lie on the causal pathway between treatment and later functioning. However, entered as a fixed covariate and it was treated as a prognostic factor rather than a mediator. No time-varying post-treatment measures were adjusted for.

To improve clarity, we have revised the Methods section to state this causal assumption and have added a directed acyclic graph (DAG) according to the reviewer's request (Figure 3, insert at p. 9), illustrating the assumed relationships between treatment, covariates, and outcome (p. 9-10).

Results:

Table 1. I find the presentation of differences between treatment groups related to the former RCT not relevant for the present study aims. I would like the Table 1 to be changed to show the results presented for the whole cohort from study inclusion, post-treatment, 4 months postpartum and 3 years postpartum, regardless of previous treatment group. This will also give the possibility to compare possible differences between the women initially included in the study and the women remaining in the study at the different time points.

Reply: *We agree that a table that shows the variables at each timepoint would be easier to interpret and as no differences between groups were identified, table 1 has been updated accordingly (p. 10).*

The initial aim of the RCT was to investigate if there were any differences between the two treatment options during the study period and the follow-ups. When the RCT was designed, a hypothesis was that maybe women could have benefits of the received treatment also in a subsequent pregnancy ant that there might be differences between the treatment groups in the long-term as one intervention was bound to clinical visits (acupuncture) and the other allowed the woman to use the TENS device at home, at her convenience.

Moreover, 60% of the included women had given birth before. To get a wider picture of the cohort I would like to know how many births they had before, not just no/yes.

Reply: *This information is added to table 1 (p. 10).*

In paragraph 2 of the Results the different PSFS activities are presented. I believe this section should be moved to the methods section. Instead, the development of PSFS-results across the study period would be presented, similar to the presentation of the PGQ-T scores.

Reply: *Yes, the paragraph regarding PSFS activities, including fig 2, has been moved into the methods section (p. /7 and the text in the Results has been revised according to your suggestion.*

Paragraph 3 of the Results. The differences between the intervention groups I found not relevant for the present study aims. Also, I wonder about the relevance to present incontinence-related symptoms.

Reply: *We agree - there is no difference between the two treatment groups at any timepoint, and reporting incontinence symptoms is not relevant for this study. The irrelevant information is removed and the text revised (p. 9).*

The repeated linear regression model:

Why calculated differences between the previous treatment groups? The interesting information is the description of the development of PSFS scores, pain-related concern and PGQ-T scores across the study period. A thought is what does the value -2.89 mean, both the negative value and then also the absolute value? From the methods section I got the information that PSFS is scored between 0 and 10. Also, regarding Table 2, what does the use of square brackets mean?

Reply: *The differences were calculated based on a hypothesis when designing the RCT that there might be differences between treatment-groups also in the long-term perspective. (Please also see our reply to your first comment. The methods section on p 9 has been revised.*
The paragraph about the repeated linear regression has been thoroughly revised to improve clarity and readability, including a revision of table 2 (p. 12).

The logistic regression analysis.
Good that the whole cohort was used in the analysis. To facilitate the interpretation the logistic regression a presentation of the 6 different pain provocation tests and the outcome (no pain/pain) could be presented in a table.

Reply: *Table 1 has been revised, and the requested information is added (p. 10).*

Discussion
My overall point of view is to present the discussion according to the STROBE checklist. I would also that the presentation of results in the discussion is used to a minimum.

Reply: *The discussion section has been revised, using the STROBE checklist and the author instructions for BMJ Open (p. 13-15).*

VERSION 2 - REVIEW

**Reviewer**	**1**
**Name**	**Magann, Everett**
**Affiliation**	**UAMS Medical Center, Obstetrics & Gynecology**
**Date**	**28-Feb-2026**
**COI**	

Changes made to this manuscript now make this study acceptable for publication

---

**Reviewer**	**2**
**Name**	**Kristiansson, Per**
**Affiliation**	**Uppsala Universitet, Public Health and Caring Sciences**
**Date**	**26-Feb-2026**
**COI**	

Review comments to the revised manuscript “Factors associated with return to meaningful activities following physiotherapy for pelvic girdle pain during pregnancy: three year follow up of a randomised controlled trial.

Here are my comments to the revisions:

In the Abstract, I would appreciate adding “113 participants at baseline”, under the Participants subtitle, to ensure consistency with the information presented in Table 1.

The newly added flow chart improves clarity and provides a helpful overview.

The statement regarding statistical adjustment is now clear and appropriate.

Table 1 has been revised and is now highly informative. However, the first sentence of the Results section refers to differences between treatment groups, which should not be linked to Table 1, as the table does not present such information.

In the first paragraph of the Results section, new sentences describe the prevalence of “symptoms”. I would prefer the term “pain localised….” Instead of symtpoms. Additionally, the method by which participants reported pain location should be described more clearly in the Methods section.

In the Methods, the PSFS-NRS outcome is described as an ordinal scale ranging from 0 to 10. If treated as an ordinal variable, PSFS-NRS should be presented as median (IQR) and analysed using methods appropriate for ordinal data. Please justify the use of linear regression model or consider revising the statistical approach. This also affects how the results should be interpreted in the Results section.

In the Methods section, a new description of how the different PSFS activities were categorised is presented. How were these categories used in the analyses?

In the Statistical analysis section, a statement about the study aim is added. From my perspective, this aim differs from the aims stated at the end of the Introduction. I apologise if my earlier comments contributed to this ambiguity, but the aims need to be aligned and clarified.

The description of covariate adjustments in the regression analysis this is now clear and accurate and the inclusion of a DAG is helpful.

Figure 4 clearly illustrates the differences in PSFS scores between baseline and the 3-year follow-up. If linear regression remains the preferred analytical approach, I would suggest presenting the results using baseline as the reference category to improve readability.

Regarding the logistic regression analysis described in the final paragraph of the Results section, why are odds ratios not reported for all three analyses presented?

Kind regards,

Per Kristiansson

Reviewer

VERSION 2 - AUTHOR RESPONSE

Reviewer: 2
Dr. Per Kristiansson, Uppsala Universitet

In the Abstract, I would appreciate adding “113 participants at baseline”, under the Participants subtitle, to ensure consistency with the information presented in Table 1.

<u>Reply:</u> This information is added on page 2.

The newly added flow chart improves clarity and provides a helpful overview.

The statement regarding statistical adjustment is now clear and appropriate.

No reply needed for these comments

Table 1 has been revised and is now highly informative. However, the first sentence of the Results section refers to differences between treatment groups, which should not be linked to Table 1, as the table does not present such information.

<u>Reply:</u> The reference to Table 1 is moved (p. 9)

In the first paragraph of the Results section, new sentences describe the prevalence of “symptoms”. I would prefer the term “pain localised….” Instead of symtpoms.

Reply: We agree that this is a better wording and requested changes have been made on p. 9

Additionally, the method by which participants reported pain location should be described more clearly in the Methods section.

<u>Reply:</u> This suggestion is addressed on p. 7.

In the Methods, the PSFS-NRS outcome is described as an ordinal scale ranging from 0 to 10. If treated as an ordinal variable, PSFS-NRS should be presented as median (IQR) and analysed using methods appropriate for ordinal data. Please justify the use of linear regression model or consider revising the statistical approach. This also affects how the results should be interpreted in the Results section.

<u>Reply:</u> Thank you for this comment. Although the PSFS items are scored on an 11-point scale (0–10), the PSFS total score in this study was calculated as the mean of the two activity ratings for each participant, consistent with previous methodological recommendations (Stratford et al., 1995; Pathak et al., 2023). When aggregated in this way, the PSFS score is commonly treated as a continuous variable in clinical research.

Linear regression models are widely used for PSFS outcomes, particularly when residual assumptions are met. In our analysis, the distribution of residuals, linearity and homoscedasticity were examined, and no violations were identified as stated in the methods section (p. 9).

In the Methods section, a new description of how the different PSFS activities were categorised is presented. How were these categories used in the analyses?

<u>Reply:</u> The categorisation of PSFS activities was performed for descriptive purposes only, in order to provide an overview of the types of activities selected by participants. Given the wide variation in the nature and intensity of these activities, the categories were not included as variables in the regression analyses. This has now been clarified in the Methods section, p. 7

In the Statistical analysis section, a statement about the study aim is added. From my perspective, this aim differs from the aims stated at the end of the Introduction. I apologise if my earlier comments contributed to this ambiguity, but the aims need to be aligned and clarified.

Reply: We agree that this statement could be misleading and it is now revised on p. 8.

The description of covariate adjustments in the regression analysis this is now clear and accurate and the inclusion of a DAG is helpful.

No reply needed for this comment

Figure 4 clearly illustrates the differences in PSFS scores between baseline and the 3-year follow-up. If linear regression remains the preferred analytical approach, I would suggest presenting the results using baseline as the reference category to improve readability.

Reply: Thank you for this suggestion. The 3-year follow-up was selected as the reference category because the primary aim of the analysis was to examine factors associated with functioning at three years postpartum. Using the 3-year follow-up as the reference allowed us to directly interpret the regression coefficients in relation to this clinically relevant endpoint. However, we agree that baseline comparisons may enhance readability and have clarified the reference category more explicitly in the Methods section (p. 8) and Results section (p. 11).

Regarding the logistic regression analysis described in the final paragraph of the Results section, why are odds ratios not reported for all three analyses presented?

Reply: Thank you for this comment. Odds ratios were reported only for the logistic regression analysis examining the association between the number of positive pelvic provocation tests and the presence of PGP at the 3-year follow-up, as this model had a binary outcome. The other analyses were conducted using linear regression models with PSFS as a continuous outcome; therefore, regression coefficients rather than odds ratios were appropriate effect measures.

-

Reviewer: 1
Prof. Everett Magann, UAMS Medical Center
Comments to the Author:
Changes made to this manuscript now make this study acceptable for publication
